# Supplementary material for: A Clinical and Genetic Evaluation of Cases with Folate Receptor α Gene Mutation: A Case Series from Türkiye
Source: Diagnostics (Basel). 2025 Apr 1;15(7):892. doi: 10.3390/diagnostics15070892 (PMC11988349; doi:10.3390/diagnostics15070892)
Supplement: Supplementary file 1 [file diagnostics-15-00892-s001.zip › diagnostics-3559145-supplementary.pdf]

Supplementary material

Supplementary material-S1: General characteristics of cases with FOLR1-associated CFD

**Case no**	1	2	3	4	5	6	7	8	9
**Family no**	1	1	1	2	3	3	4	5	6
**Gender**	F	F	F	F	F	M	F	M	F
**Consanguinity**	+	+	+	-	+	+	+	+	+
**Age of first complaint (year)**	0,66	2,5	5,5	4	6	3	3	4	5
**First complaint**	Inability to sit or crawl	Febrile convulsion	Difficulty going up and down stairs	Hyperactivity and learning disabilities	Difficulty walking	Speech delay	Speech delay (inability to speak fluently)	Autistic findings	Febrile convulsion
**Age of diagnosis**	16.5	11.5	5.5	23.5	13	6	4.6	5	8.5
**Age at first seizure (year)**	1.5	2.5		11	9	4	4	5	5
**Variant**	c.610C>T (p.R204Ter) homozygous	c.610C>T (p.R204Ter) homozygous	c.610C>T (p.R204Ter) homozygous	c.610C>T (p.R204Ter) homozygous	c.610C>T (p.R204Ter) homozygous	c.610C>T (p.R204Ter) homozygous	c.610C>T (p.R204Ter) homozygous	c.466T>G (p.W156G) homozygous	c.591C>A (p.W197Ter) homozygous
**EEG**	Sharp wave and spike wave activity in the frontal, fronto-central, and temporo-occipital regions of both hemispheres	Sharp wave and sharp slow wave activity originating from the frontal, temporo-occipital, fronto-temporal, fronto-central, and temporal regions of both hemispheres was observed	N/A (There was no history of seizures)	Epileptic activity and 3-4 Hz subcortical discharges in both temporo-parietal regions	In the left hemisphere, 2 Hz spike-and-slow wave activity originating from the temporal, temporo-occipital, and fronto-central regions, along with sharp waves, spikes, and multifocal spike-and-slow waves were observed. In the right hemisphere, similar activity was observed in the frontal, fronto-central, and temporal regions. (LGS?)	Generalized 1-2.5 Hz spike-and-slow wave and multiple spike-wave discharges	Epileptiform abnormality originating from the anterior regions of the hemispheres and becoming generalized	Low-amplitude sharp-slow wave activity, showing synchronous generalization in the left frontal and right centroparietal regions, was frequently observed	N/A
**EMG**	Widespread polyneuropathy with predominantly	Widespread polyneuropathy with predominantly	Normal EMG	Widespread sensorimotor polyneuropathy	N/A	N/A	Normal EMG	N/A	N/A

	motor axonal damage	motor axonal damage		with axonal damage					
**Folate (ng/ml)**	3.5	3.2	4	2.7	3.3	3.8	3	3.7	1.3
**Homocysteine (µmol/l)**	N/A	20.9	14.2	18.1	3.6	5	9.4	6.8	26.9
**Hemoglobin (gr/dl)**	11.2	12.6	13.7	9.7	11.4	11.7	13.9	12.5	13.4
**MCV (fL)**	76.2	87.6	80.8	76.9	94.1	81.4	81.7	82.8	85
**Brain MRI**	Cerebral atrophy, cerebellar atrophy, supratentorial hypomyelination	Cerebral atrophy, cerebellar atrophy, supratentorial hypomyelination, diffuse thickening of the calvarium	Diffuse patchy hypomyelinated areas are observed in the deep and subcortical white matter of both cerebral hemispheres, more prominent in the parietal lobes	Cerebral atrophy, cerebellar atrophy, supratentorial hypomyelination, calcification in the basal ganglia	Cerebral atrophy, cerebellar atrophy, supratentorial hypomyelination	Cerebellar atrophy, hypomyelination	Hypomyelination, focal atrophy of the corpus callosum body, arachnoid cyst	Millimetric patchy lesions were observed in the periventricular area, bilateral frontal, parietal, and occipital lobes, particularly in the pericallosal region and with a perpendicular orientation to the corpus callosum	Mild cerebellar atrophy, areas of encephalomalacia in the parieto occipitotemporal area
**Brain MRS**	N/A	N/A	N/A	Normal	N/A	N/A	Normal	Minimal increase in choline peak, minimal decrease in inositol peak	Normal
**Age of starting folinic acid (year)**	N/A	12	5.5	23.5	13	5.8	4.6	6.2	8.2
**Oral folinic acid dosage (mg/kg/day)**	N/A	5.4	5.5	3	4.6	6	5.2	5	7.5
**IV folinic acid dosage**	100 mg/day	100 mg/week	100 mg/week	200 mg/week	100 mg/week	100 mg/week	100 mg/week	25 mg/kg/month	N/A
**Physio- therapy**	N/A	+	N/A	+	+	+	N/A	+	N/A
**VNS**	N/A	N/A	N/A	N/A	+	+	N/A	N/A	N/A
**Antiepileptic drugs**	Valproic acid, topiramate, clonazepam	Clonazepam, valproic acid, clobazam, levetiracetam		Lamotrigine, valproic acid, levetiracetam, lacosamide	Valproic acid, lamotrigine, rufinamidum, clonazepam, VNS	Valproic acid, lamotrigine, rufinamidum, clonazepam, VNS	Valproic acid, clobazam	Clonozepam, valproic acid, topiramate	Levetiracetam, carbamazepine

Abbreviations: F: Female, **M:** Male, **N/A:** Not applicable, **EEG:** Electroencephalography, **EMG:** Electromyography, **MCV:** Mean Corpuscular Volume, **MRI**: Magnetic Resonance Imaging, **MRS:** Magnetic Resonance Spectroscopy, **VNS:** Vagus Nerve Stimulation
